# Editorial: The role of enhancers in cancer

**DOI:** 10.3389/fcell.2025.1550404

**Published:** 2025-06-17

**Authors:** Sonia V. Forcales

**Affiliations:** ^1^ Serra Húnter Programme, Immunology Unit, Department of Pathology and Experimental Therapy, School of Medicine, Universitat de Barcelona, L'Hospitalet de Llobregat, Spain; ^2^ Immunity, Inflammation and Cancer Group, Oncobell Program, Institut d'Investigació Biomèdica de Bellvitge-IDIBELL, L'Hospitalet de Llobregat, Spain

**Keywords:** enhancers, transcription, gene expression, chromatin, cancer

As the field of cancer biology advances, enhancers have emerged as master regulators, fine-tuning gene expression programs that dictate cell fate and transformation. This Research Topic gathers several works revealing how enhancer dysfunction can fuel oncogenic programs that drive tumor initiation and progression. While enhancers are at the core, distinct mechanisms promote their oncogenic potential. For instance, structural variants, including chromosomal rearrangements, duplications, and translocations, can trigger “enhancer hijacking” repositioning active enhancers near proto-oncogenes and leading to their aberrant activation. This mechanism has been implicated in multiple cancers, from medulloblastoma to leukemia, underscoring the critical role of enhancer mis-regulation in tumorigenesis. Davidson et al. investigate this phenomenon by examining the MYEOV locus, an enhancer-associated region that plays a regulatory role in CCND1 expression. Their findings suggest that MYEOV originated as an enhancer long before acquiring proto-oncogenic properties and continues to function as a key regulator in cancers where MYEOV and CCND1 are co-amplified. This study shows how enhancers can transition from normal regulatory elements to oncogenic drivers over time, and suggests that targeting their activity could offer new therapeutic opportunities. Complementing this work, Della Chiara et al. provide an overview of current research on enhancer hijacking, discussing how 3D genome architecture rearrangements can disrupt enhancer-promoter interactions across various cancers. By investigating structural alterations affecting topologically associating domains (TADs), they illustrate how these changes drive oncogene activation, using cases such as the TMPRSS2-ERG fusion in prostate cancer and NSD2 activation in multiple myeloma. Their review also highlights novel computational tools, such as NeoLoopFinder and the Activity-By-Contact (ABC) model, which enhance our ability to detect enhancer hijacking events and identify new cancer drivers.

Other mechanisms highlight the role of transcription factor (TF) regulatory networks in cancer, where enhancers serve as key hubs orchestrating gene expression programs. Yan et al. construct a TF-enhancer-target regulatory network in hepatocellular carcinoma (HCC), identifying DAPK1 as a core regulatory gene. Their findings reveal that enhancer-driven transcriptional programs in HCC are intricately linked to immune pathways, positioning DAPK1 as a promising biomarker and therapeutic target for immune-based therapies in liver cancer. In the context of triple-negative breast cancer (TNBC), one of the most aggressive and treatment-resistant breast cancer subtypes, Shi et al. explore enhancer-driven transcriptional regulatory circuits and identify USF1, SOX4, and MYBL2 as TNBC-specific core TFs that drive enhancer-promoter interactions and reprogram gene expression. Their findings suggest that these circuits contribute to TNBC’s aggressive phenotype and therapy resistance, offering potential new targets for intervention. Together, these studies underscore the importance of enhancer-mediated TF networks in shaping tumor progression and immune responses, providing valuable insights for targeted therapies.

A different dimension of enhancer function is explored by Long et al., who investigate the enhancer-like activity of the long non-coding RNA MALAT1 in intestinal tumorigenesis. Their study reveals that MALAT1 regulates transcription and alternative splicing of epithelial genes involved in microbial responses, in part through direct chromatin interactions. Using a mouse model of familial adenomatous polyposis, they demonstrate that MALAT1 deficiency increases polyp formation, although polyp size is reduced, suggesting a dual role in both tumor initiation and progression. Among the 30 MALAT1-regulated targets shared across the small intestine and colon, ZNF638 and SENP8 emerged as key genes positively regulated by MALAT1, with direct chromatin occupancy at their loci. Notably, high expression of ZNF638 and SENP8 correlates with improved overall and disease-free survival in colon adenocarcinoma, supporting a tumor-suppressive function for MALAT1 in maintaining genes with favorable prognostic value. These findings broaden our understanding of enhancer-like regulation by lncRNAs and underscore MALAT1’s potential as a biomarker and therapeutic target in colorectal cancer.

Together, these studies reinforce the central role of enhancers in shaping cancer-associated gene networks and genome architecture. By dissecting enhancer mechanisms across different cancers, we gain deeper insights into the interplay between genome structure and gene expression. As computational tools and analytical models advance, our ability to decode enhancer hijacking and other forms of enhancer dysregulation will improve, paving the way for targeted therapeutic strategies. In summary, enhancer biology sits at the crossroads of oncogenic regulation and therapeutic innovation. As we continue to refine our understanding and develop more sophisticated methodologies, the potential to harness enhancers for cancer diagnosis and treatment becomes increasingly feasible. These insights bring us closer to the goal of precision medicine, where targeting the unique regulatory landscapes of individual cancers holds the promise of transforming patient care.

